# A multicenter prospective cohort study evaluating 30-day outcomes after an emergency department visit for hyperglycemia in Canadian adults with type 1 or 2 diabetes

**DOI:** 10.1007/s43678-025-00913-6

**Published:** 2025-04-25

**Authors:** Justin W. Yan, Kristine Van Aarsen, Joe Thorne, Igor Karp, Tamara Spaic, Selina L. Liu, Nicolas Woods, Ian G. Stiell, For the Network of Canadian Emergency Researchers (NCER)

**Affiliations:** 1 Division of Emergency Medicine, Department of Medicine, Schulich School of Medicine and Dentistry, Western University, London, ON Canada; 2 Lawson Health Research Institute, London, ON Canada; 3 Department of Epidemiology and Biostatistics, Schulich School of Medicine and Dentistry, Western University, London, ON Canada; 4 Division of Endocrinology and Metabolism, Department of Medicine, Schulich School of Medicine and Dentistry, Western University, London, ON Canada; 5 Department of Emergency Medicine, University of Ottawa, Ottawa, ON Canada; 6 Ottawa Hospital Research Institute, Ottawa, ON Canada; 7 LHSC Victoria Hospital, London, ON Canada

**Keywords:** Emergency department, Hyperglycemia, Diabetes, Patient outcomes, Services d’urgence, Hyperglycémie, Diabète, Résultats des patients

## Abstract

**Objectives:**

Previous retrospective studies have demonstrated that patients with sub-optimally controlled diabetes have higher healthcare resource utilization in emergency department (ED) management of hyperglycemia compared to those with good glycemic control. This study’s objective was to prospectively describe 30-day outcomes including return visits and hospitalizations after an initial ED visit for hyperglycemia.

**Methods:**

We conducted a multicenter prospective cohort study of adults ≥ 18 years at four Ontario academic EDs diagnosed with hyperglycemia, diabetic ketoacidosis, or hyperosmolar hyperglycemic state. The primary outcome was an unplanned repeat ED visit for hyperglycemia within 30 days of index visit. We conducted telephone follow-up at 14 and 30 days to determine additional outcomes. Data were summarized using descriptive statistics.

**Results:**

There were 657 ED visits for hyperglycemia representing 594 unique patients. Mean (SD) age was 52.0 (18.2) years, 53.2% were male. Within 30 days, 96 (14.7%) had a return ED visit for hyperglycemia, 49 (7.5%) were hospitalized, and 4 (0.6%) died. We were able to contact 383 (58.3%) patients by telephone at 14 days and 275 (41.9%) at 30 days. Of these, 68.3% self-identified as Caucasian/White, while 6.3% were Indigenous. 44.9% reported an annual household income below $50,000. 29.1% of working patients took time off following their index visit.

**Conclusion:**

This prospective study describes 30-day outcomes and healthcare utilization of ED patients presenting for hyperglycemia. ED clinicians should be aware of the potential for subsequent healthcare utilization and risk for readmission and intervene as appropriate to reduce adverse outcomes in patients with diabetes presenting with hyperglycemia.

## Résumé

**Objectifs:**

Des études rétrospectives antérieures ont démontré que les patients atteints d’un diabète sous-optimal avaient une utilisation plus élevée des ressources de soins dans la gestion de l’hyperglycémie aux urgences (DE) comparativement à ceux qui avaient un bon contrôle glycémique. L’objectif de cette étude était de décrire prospectivement les résultats après 30 jours, y compris les visites de retour et les hospitalisations après une première visite d’urgence pour cause d’hyperglycémie.

**Méthodes:**

Nous avons mené une étude de cohorte prospective multicentrique sur des adultes âgés de 18 ans dans quatre centres universitaires de l’Ontario diagnostiqués d’hyperglycémie, de kétoacidose diabétique ou d’état hyperosmolaire hyperglycémique. Le résultat principal était une visite non planifiée de réadmission pour l’hyperglycémie dans les 30 jours suivant la visite d’index. Nous avons effectué un suivi téléphonique à 14 et 30 jours pour déterminer d’autres résultats. Les données ont été résumées au moyen de statistiques descriptives.

**Résultats:**

Il y a eu 657 visites en salle d’opération pour l’hyperglycémie représentant 594 patients uniques. L’âge moyen (SD) était de 52,0 (18,2) ans, 53,2 % étaient des hommes. Dans les 30 jours, 96 (14,7 %) ont eu une visite de réadmission pour hyperglycémie, 49 (7,5 %) ont été hospitalisés et 4 (0,6 %) sont décédés. Nous avons pu contacter 383 (58,3 %) patients par téléphone à 14 jours et 275 (41,9 %) à 30 jours. Parmi ceux-ci, 68,3 % se sont identifiés comme étant de race blanche et 6,3 % étaient autochtones. 44,9 % ont déclaré un revenu annuel du ménage inférieur à 50 000 $. 29,1 % des patients ayant travaillé ont pris un congé après leur visite de l’index.

**Conclusions:**

Cette étude prospective décrit les résultats sur 30 jours et l’utilisation des soins de santé chez les patients présentant une hyperglycémie. Les cliniciens de la DE doivent être conscients du potentiel d’utilisation ultérieure des soins de santé et du risque de réadmission, et intervenir au besoin pour réduire les résultats indésirables chez les patients diabétiques présentant une hyperglycémie.

## Clinician’s capsule

**Table:** 

***What is known about the topic?***
Previous studies have shown that patients with suboptimally-controlled diabetes use more healthcare resources compared to those with good glycemic control.
***What did this study ask?***
This study aimed to prospectively describe 30-day outcomes including return visits and hospitalizations after an initial ED visit for hyperglycemia.
***What did this study find?***
Within 30 days, 14.7% of patients had a return ED visit for hyperglycemia,7.5% were hospitalized, and 0.6% died.
***Why does this study matter to clinicians?***
Clinicians should be aware of the risk for readmission and intervene to reduce adverse outcomes in patients presenting with hyperglycemia.

## Introduction

Diabetes mellitus is one of the most prevalent chronic diseases in Canada with an estimated prevalence of 10% (> 3.7 million people) [1]. Over 40% of Canadian healthcare system funds spent on diabetes care (~ $6.6 billion) is due to acute hospitalizations and ED visits [2], and patients with sub-optimally controlled diabetes may have recurrent ED presentations for hyperglycemia. It has previously been shown that almost 20% of patients had an unplanned return ED visit for hyperglycemia within 30 days of an initial hyperglycemia presentation, and almost half of these return visits led to hospital admission [3].

Past retrospective studies, and a systematic review performed by our research group, have described adverse outcomes after an ED visit for hyperglycemia and attempted to identify predictors of recurrent unplanned ED visits [4–6]. However, conclusions from these studies are limited due to the inherent weaknesses associated with health records reviews and/or systematic reviews. Furthermore, while the focus of previous studies has been on healthcare system-based outcomes, prospective data collection on additional outcomes deemed important to patients according to direct input from patient partners with lived experience (including missed time off school or work, medication adjustments, and further healthcare provider visits) is lacking. Finally, none of the previous studies reported data on race and socioeconomic status, which are important determinants of healthcare utilization and outcomes, as these are not routinely collected in Canadian healthcare systems and are underreported in Canadian research [7, 8]. Therefore, the need for a prospective study to report these data to describe this important and growing population is essential.

The objective of this multicenter study was to prospectively describe 30-day outcomes including return visits and hospitalizations after an initial ED visit for hyperglycemia in patients with type 1 or 2 diabetes. In addition, we sought to document demographic information including race and socioeconomic status and describe patient-important outcomes, including missed time off school or work and healthcare provider follow-up after the initial ED hyperglycemia visit.

## Methods

### Study design, time period, and setting

This was a multicenter prospective cohort study of eligible adult patients with an ED diagnosis of hyperglycemia, diabetic ketoacidosis, or hyperosmolar hyperglycemic state. The setting was the EDs of two large, tertiary care, academic centers in Ontario comprising four sites (London Health Sciences Centre’s Victoria and University Campuses and The Ottawa Hospital’s Civic and General Campuses) with a combined census of > 300,000 visits annually. Enrollment occurred from 2017 to 2020 at London Health Sciences Centre until all research activities paused due to the COVID-19 pandemic in March 2020 but resumed in 2022. The Ottawa Hospital sites enrolled patients from December 2022–November 2023. Our protocol was approved by Western University’s Health Sciences Research Ethics Board (#107826) and the Ottawa Health Science Network Research Ethics Board (20190615-01H).

### Population

All initial (index) ED visits of adult (≥ 18 years) patients with either a known or unknown history of diabetes mellitus and a diagnosis of hyperglycemia (> 11.0 mmol/L), diabetic ketoacidosis, or hyperosmolar hyperglycemic state were eligible to be enrolled. This included patients with either type 1 or type 2 diabetes, either admitted or discharged from the ED, and with or without co-morbid diagnoses in addition to hyperglycemia (e.g., infection, cardiac ischemia, etc). We excluded patients who were initially assessed at a peripheral hospital and subsequently transferred to our sites for ongoing management by an inpatient service, and those who were unable (or did not have an available substitute decision-maker) to provide consent.

### Informed consent process

Informed consent was required for patients to be contacted for telephone follow-up at all sites. At London Health Sciences Centre, trained research assistants used the ED tracking system to screen all potentially eligible patients with a reason for visit that could be related to hyperglycemia (e.g., nausea/vomiting, abdominal pain, “high blood sugar”) as well as laboratory values and if eligible, patients were flagged for a member of the circle of care to obtain consent. Emergency physicians confirmed enrollment eligibility and obtained consent for the research team to contact the patient by telephone at 14- and 30-day post-ED visit. At The Ottawa Hospital, research assistants screened daily logs of all ED visits including appropriate discharge diagnoses for eligible participants, and those who had previously consented to be contacted for research purposes according to the flag in the electronic medical record were contacted for follow-up. Follow-up was limited to English-speaking patients.

### Data collection, outcomes measures, and data analysis

For each enrolled patient, data pertaining to their ED visit were abstracted by research personnel from the local site’s electronic medical record into a study-specific REDCap case record form, which consisted of six data instruments for the health records data abstraction (demographics, medical history, visit history, ED variables, diagnostic variables, and discharge variables including hospital-based outcomes and follow-up) and two data instruments for the telephone follow-ups.

Research personnel performed a detailed review of patients’ medical record at 30 days to determine if they had subsequent hyperglycemia-related ED visits, hospital or intensive care unit admissions, or died. At London Health Sciences Centre, the electronic medical record is integrated with 13 surrounding peripheral EDs, so hospital-based outcomes (return visits and admissions) within the catchment area were accurately captured. At The Ottawa Hospital, the electronic medical record is not linked with peripheral hospitals. Research assistants contacted patients at 14 and 30 days via telephone for follow-up data regarding additional outcomes, including repeat visits to see a healthcare provider outside our sites, changes in diabetic medications, and time taken off work or school. Further characteristics including self-reported ethnicity, socioeconomic status, and education level were also asked about on follow-up as this information is not collected in the electronic medical record. Patients unable to be reached after three attempts over three different days were declared lost to follow-up.

Consistent with previous studies [4–6], our primary outcome was an unplanned return visit to the ED for hyperglycemia within 30 days of the index visit. Secondary outcomes included unplanned admission to hospital for hyperglycemia, intensive care unit admission, and death from any cause within 30 days. Finally, our 14- and 30-day telephone follow-up determined patient-reported outcomes that we could not obtain from medical record review, including non-hospital-based healthcare utilization (e.g., visits to family physician or other healthcare provider for diabetes care, time taken off school or work, and medication changes).

The distributions of relevant patient characteristics were summarized by calculating descriptive statistics (means and standard deviations with 95% confidence intervals (CI) or medians and interquartile ranges (IQR) for quantitative characteristics and percentages for categorical characteristics).

## Results

There were 657 ED visits for hyperglycemia representing 594 unique patients enrolled in the study. Individual patient characteristics are summarized in Table 1. Overall, 53.2% were male, mean (SD) age was 52.0 (18.2) years, and 50.8% were on insulin. Over half (57.6%) of the patients had known type 2 diabetes, 24.9% had known type 1 diabetes, 57.7% were followed by a family physician, and 29.6% by an endocrinologist.

**Table 1:** Characteristics of 594 unique emergency department patients presenting for hyperglycemia

Patient characteristic	*n* = 594
Sex (%)	
Male	316 (53.2)
Female	276 (46.5)
Intersex	2 (0.3)
Mean age, years (SD)	52.0 (18.2)
Range	18–96
Residence in a nursing home or long-term care facility (%)	16 (2.7)
No fixed address	10 (1.7)
Previously known history of diabetes (%)	
Type 1	148 (24.9)
Type 2	342 (57.6)
New diagnosis/no documented history of diabetes	98 (16.5)
Unclear	4 (0.7)
Gestational	1 (0.2)
Use of diabetic medications prior to index emergency visit (%)	
Insulin	302 (50.8)
Oral hypoglycemic	273 (46.0)
None	138 (23.2)
Followed by (%)	
Family physician	343 (57.7)
Endocrinology	176 (29.6)
Diabetes education nurse	59 (9.9)
Internal medicine	53 (8.9)
Unknown/not documented	166 (27.9)
Comorbidities (%)	
Hypertension	295 (49.7)
Hyperlipidemia	192 (32.3)
Psychiatric illness	157 (26.4)
Coronary artery disease	90 (15.2)
Chronic obstructive pulmonary disease/asthma	75 (12.6)
Cancer	68 (11.5)
Stroke/transient ischemic attack	57 (9.6)
Chronic renal failure	28 (4.7)
Peripheral vascular disease	17 (2.9)
Dementia	13 (2.2)
None of the above	162 (27.3)

*SD* standard deviation

Characteristics of all 657 ED visits for hyperglycemia are summarized in Table 2. The mean (SD) initial capillary blood glucose level with pre-hospital emergency medical services was 24.4 (7.4) mmol/L. The most common reason for visit was “high blood sugar” (55.3%), followed by “fatigue/weak/dizzy” (11.7%) and nausea/vomiting (10.0%). The final ED diagnosis was “hyperglycemia/diabetes” in 70.9%, diabetic ketoacidosis in 20.4%, and hyperosmolar hyperglycemic state in 4.1% of patients (Table 3). There were 224 (34.1%) patients admitted to hospital, 212 to the ward and 12 to the intensive care unit. The median (IQR) hospital length of stay was 3.0 (4.0) days.

**Table 2:** Characteristics of all 657 emergency department visits for hyperglycemia

Characteristic	*n* = 657 (%)
Study site	
London Health Sciences Centre—Victoria Campus	278 (42.3)
London Health Sciences Centre—University Campus	188 (28.6)
The Ottawa Hospital—General Campus	96 (14.6)
The Ottawa Hospital—Civic Campus	95 (14.5)
Arrival mode	
Self	443 (67.4)
EMS	213 (32.4)
Police	1 (0.2)
CTAS (*n* = 656)	
1	10 (1.5)
2	343 (52.3)
3	283 (43.1)
4	19 (2.9)
5	1 (0.2)
EMS blood glucose, mmol/L **(*n* = 88)** (mean, SD)	24.4 (7.4)
Chief complaint	
High blood sugar	363 (55.3)
Fatigue/weakness/dizzy	77 (11.7)
Nausea/vomiting	66 (10.0)
Abdominal pain	29 (4.4)
Chest pain	21 (3.2)
Shortness of breath	16 (2.4)
Other	92 (14.0)
Sentinel visit for any reason within previous 14 days	69 (10.5)

Bold indicates that not all participants had data for all variables

*EMS* emergency medical services, *CTAS* Canadian Triage and Acuity Scale, *SD* standard deviation

**Table 3:** 30-day outcomes, final diagnoses, consultations, and disposition for emergency department hyperglycemia visits (*n* = 657)

30-day outcomes	
Return visit to ED for hyperglycemia	94 (14.3)
Hospital admission for hyperglycemia	41 (6.2)
Intensive care unit admission for hyperglycemia	0 (0.0)
Death (any cause) within 30 days	4 (0.6)
Final hyperglycemic diagnosis	
Hyperglycemia or diabetes	466 (70.9)
Diabetic ketoacidosis	134 (20.4)
Hyperosmolar hyperglycemic state	27 (4.1)
Other (e.g., cellulitis, diabetic gastroparesis, etc., or missing diagnosis)	30 (4.6)
Consultations in ED*	
Internal medicine	198 (30.1)
Endocrinology	45 (6.8)
Intensive care unit	11 (1.7)
Other (general surgery, cardiology, urology, respirology, etc.)	91 (13.9)
No documented consultation	403 (61.3)
Disposition from ED	
Discharged	428 (65.1)
Admitted	224 (34.1)
To ward	212 (32.3)
To intensive care unit	12 (1.8)
Median (IQR length of stay, days **(*n* = 223)**	3.0 (4.0)
Left against medical advice	5 (0.8)
Death in ED	0 (0)
Death in hospital, after admission	3 (1.4)

Bold indicates that not all participants had data for all variables

*ED* emergency department, *IQR* interquartile range

^*^Patients may have had more than one consultation in the ED

Upon review at 30 days, 94 (14.3%) patients had an unplanned return ED visit and 41 (6.2%) were subsequently hospitalized for hyperglycemia (Table 3). Four (0.6%) patients died within 30 days.

We were able to contact 383 patients (58.3% of all 657 ED visits, 66.1% of 579 unique individuals) for telephone follow-up at 14 days. There were no differences between those with follow-up data and those lost to follow-up in age, sex, type of diabetes, or use of insulin, but there was a higher proportion with no fixed address (*p* < 0.001) and not followed by healthcare providers for diabetes (*p* < 0.001) in the lost to follow-up group. Of the visits, 110 (28.8%) patients had follow-up with their family physician, and 78 (20.4%) had follow-up with their endocrinologist (Table 4). A further 18 (4.7%) reported seeing another ED and 35 (9.2%) reported hospitalization for a median (IQR) duration of 4.0 (5.0) days. Sixty-four of two hundred twenty (29.1%) working patients reported the need to take time off work for a mean (SD) of 7.3 (6.0) days. Of individuals reporting ethnicity or racial heritage, the majority (68.3%) self-identified as Caucasian/White and 6.3% as Indigenous (Table 1). Of the 285 who provided income data, 94 (33.0%) reported an approximate annual household income < $25,000 and 78 (27.4%) from $25,000–$49,999.

**Table 4:** Patient-reported outcomes upon telephone follow-up completed at 14 days (*n* = 383) and 30 days (*n* = 275)

14-day telephone follow-up completed	*n* = 383 (%)
Time taken off work (*n* = 380*)	
Not applicable/not working	160 (42.1)
No	156 (41.1)
Yes	64 (16.8)
Mean number of days (SD)	7.3 (6.0)
Diabetes medication adjustments since ED visit (*n* = 381*)	
Insulin	117 (30.7)
Increased dose	59 (50.9)
Decreased dose	10 (8.6)
Started	47 (40.5)
Diabetes pills	92 (24.1)
Increased dose	19 (20.9)
Decreased dose	14 (15.4)
Changed type	8 (8.8)
Started	50 (54.9)
No changes	201 (52.8)
Follow-up with another physician post-ED visit (*n* = 382*)	
Family physician	110 (28.8)
Endocrinologist	78 (20.4)
Internal medicine	32 (8.4)
Another ED physician	18 (4.7)
Walk-in clinic	2 (0.5)
Other	21 (5.5)
No physician follow-up since discharge	163 (42.7)
Admitted to another hospital since ED visit (*n* = 382*)	
Yes	35 (9.2)
No	347 (90.8)
Median length of stay (IQR)	4.0 (5.0)
Highest level of education completed (*n* = 382*)	
Grade 8/elementary	53 (13.9)
Secondary school	125 (32.7)
College diploma	111 (29.1)
Bachelor’s degree	48 (12.6)
Graduate or professional degree	18 (4.7)
No schooling	4 (1.0)
Prefer not to say	23 (6.0)
Ethnicity or racial heritage (*n* = 382*)	
White/Caucasian	261 (68.3)
Indigenous	24 (6.3)
Middle Eastern or Arabic	15 (3.9)
Black or African Canadian	15 (3.9)
South Asian	8 (2.1)
Other	27 (7.1)
Prefer not to say	32 (8.4)
Approximate household annual income **(*n* = 383)**	
Prefer not to say	98 (25.6)
Responded **(*n* = 285)**	
Less than $25,000	94 (33.0)
$25,000 to $49,999	78 (27.4)
$50,000 to $74,999	49 (17.2)
$75,000 to $99,999	24 (8.4)
$100,000 or more	40 (14.0)

30-day telephone follow-up completed	***n* = 275 (%)**
Return to another ED for hyperglycemia (*n* = 274*)	
Yes	7 (2.6)
No	267 (97.4)
Admitted to another hospital since ED visit for any reason	
Yes	20 (7.3)
No	255 (92.7)
Median length of stay (IQR)	4.0 (2.8)

Bold indicates that not all participants had data for all variables

*ED* emergency department, *SD* standard deviation, *IQR* interquartile range

^*^Some patients did not respond to question

At 30 days, we reached 275 (41.9%) patients for follow-up, and of these, 20 (7.3%) reported hospitalization for any reason for a median (IQR) duration of 4.0 (2.8) days.

## Discussion

### Interpretation of findings

In this study, we describe 14- and 30-day outcomes after an initial ED visit for hyperglycemia and subsequent healthcare system resource utilization including return ED visits, hospitalizations, and follow-up. In addition, we report demographic information including ethnicity and socioeconomic status, and describe specific patient-important outcomes such as missed time off work in a subset of this population. To our knowledge, this is the first Canadian multicenter prospective cohort study examining ED visits for hyperglycemia in patients with diabetes and their clinical and additional patient-important outcomes.

### Comparison to previous studies

Our study adds to the body of literature investigating this important population and used the same primary outcome as past studies on this topic (i.e., unplanned return ED visit for hyperglycemia within 30 days of the index visit) [4–6]. We found a similar frequency of the primary outcome, with 14.3% in this study compared to 18.7% in a previous health records review done at our centers [4]. Frequency of hospitalization at 30 days between both studies was also similar (6.2% in this study versus 8.8% in the previous study). In addition, a recent population-based study conducted by our study group examined ED visits for hyperglycemia over a 10-year period (2010–2020) and found a primary outcome rate of 11.0% [6]. In the present study, our reported rate of 14.3% may be an underestimate of the primary outcome as there may have been patients who had subsequent ED visits for hyperglycemia whom we were unable to reach by telephone, and some patients who declined enrollment may have been more likely to have return ED visits. Nonetheless, it is important for clinicians to realize that patients presenting to the ED for hyperglycemia are at co-risk of returning or clinically deteriorating such that they subsequently require hospital admission.

Multiple previous studies have described the independent association between lower socioeconomic status and higher rates of both ED visits and re-hospitalization after an ED visit for a variety of healthcare conditions both in Canada and internationally [9–12]. However, very few studies—especially in Canada—have examined socioeconomic status and diabetes- or hyperglycemia-related ED visits or hospitalizations. One older population-based cohort study examined healthcare system claims from 1992 to 1999 and found an association between lower income level and hospitalizations or ED visits for hyperglycemia or hypoglycemia in Ontario [13]. This association persisted after adjusting for multiple variables including age, sex, urban versus rural residence, comorbidity, frequency of physician visits, continuity of care, physician specialty, and geographic region. In contrast, admission rates for other non–ambulatory care–sensitive conditions (i.e., appendicitis and hip fracture) were not associated with socioeconomic status. A more recent population-based cohort study also done in Ontario examined ED visits for hyperglycemia from 2010 to 2020 and found that residing in regions with fewer visible minority groups and with less education or employment was independently associated with recurrent unplanned ED visits for hyperglycemia at 30 days [6]. Several U.S.-based studies have examined the effect of race and ethnicity on healthcare utilization and found that Black/African American adults had approximately three times higher diabetes-specific ED visits compared to White/Caucasian individuals [14, 15]. However, very little on racial or ethnic disparities has been reported in a Canadian context. While we could not examine race/ethnicity or socioeconomic status as independent predictors for adverse events due to low outcome rates, we found that over two-thirds of individuals in whom follow-up contact was made self-identified as White/Caucasian, and over 60% of those reporting income data had a self-reported annual household income less than $50,000.

### Strengths and limitations

Our study’s strengths include its multicenter prospective design involving telephone follow-up, which enabled us to capture data not routinely collected in health records reviews, including resource utilization outside our hospital systems and socioeconomic status, race, and education data. However, our study had several limitations. Enrolment and follow-up rates were lower than expected, which we hypothesize may be partly attributed to the COVID-19 pandemic given the decrease in total ED visits and decrease in hospital staff and patient participation during part of our study period. The pandemic also delayed start-up at two EDs, while enrollment was stopped and restarted at the others. Furthermore, enrollment may have been limited by selection bias, as consent was required to contact patients for follow-up and the consent and follow-up processes were both conducted in English. This may have led to under-representation of racial and ethnic minorities, although the 2021 Canadian census reports almost 70% of Canada self-identified as White/Caucasian [16], similar to the 68.3% in our study. The use of telephone follow-up would have excluded patients without access to telephones, which could also skew the results towards individuals of higher socioeconomic status. In addition, outcome rates reported in our study may be an underestimate of the true incidence if patients presented to centers that were not linked to the electronic medical record of our hospital systems or did not report outcomes upon follow-up. Finally, our findings may not be generalizable to other jurisdictions, especially those outside of Canada, where there is not a public, single-payer, universal healthcare system.

### Clinical implications

Hyperglycemia is a common presentation to the ED and its frequency will only increase as the prevalence of diabetes in our population increases. Our study prospectively confirms that patients with suboptimal glycemic control are at risk for unplanned return visits to the ED and may require subsequent hospitalization. Of the participants reporting income data, over 60% had an annual household income less than $50,000, which is consistent with other studies suggesting that lower socioeconomic status is associated with higher ED utilization.

### Research implications

Future studies could explore accurate identification of ED patients presenting with hyperglycemia at higher risk of adverse outcomes and study interventions to reduce healthcare resource utilization in this population. In addition, studies might focus on comparing outcomes by sociodemographic factors such as gender or sex, race, age, or socioeconomic status, and investigating regional variations in outcomes.

## Conclusion

In conclusion, this prospective study describes 30-day outcomes including recurrent ED visits and subsequent hospitalization of patients presenting to the ED for hyperglycemia. We also describe socioeconomic and racial/ethnic demographics and patient-important outcomes of this important population. ED clinicians need to be aware of the potential for subsequent healthcare utilization and risk for readmission and intervene as appropriate to reduce adverse outcomes in patients with diabetes presenting with hyperglycemia.

## Data Availability

Data generated or analyzed during this study are available from the corresponding author upon reasonable request.
